# How to Assess Oral Narrative Skills of Children and Adolescents with Intellectual Disabilities: A Systematic Review

**DOI:** 10.3390/bs14040308

**Published:** 2024-04-10

**Authors:** Victoria Sánchez-Gómez, Miguel Ángel Verdugo, María Isabel Calvo, Antonio M. Amor, Blanca Palomero-Sierra, Laura Zampini

**Affiliations:** 1 Institute for Community Inclusion (INICO), University of Salamanca, 37005 Salamanca, Spain; verdugo@usal.es (M.Á.V.); isabelc@usal.es (M.I.C.); aamor@usal.es (A.M.A.); bpalomeros@usal.es (B.P.-S.); 2 Department of Personality, Assessment, and Psychological Treatments, University of Salamanca, 37005 Salamanca, Spain; 3 Department of Didactics, Organization, and Research Methods, University of Salamanca, 37008 Salamanca, Spain; 4 Department of Basic Psychology, Psychobiology, and Methodology of Behavioral Science, University of Salamanca, 37005 Salamanca, Spain; 5 Department of Psychology, University of Milano-Bicocca, 20126 Milan, Italy; laura.zampini1@unimib.it

**Keywords:** narrative competence, narrative skills, intellectual disability, assessment, validity, reliability

## Abstract

Children and adolescents with intellectual disabilities (ID) often encounter difficulties with narrative skills. Yet, there is a lack of research focusing on how to assess these skills in this population. This study offers an overview of the tools used for assessing oral narrative skills in children and adolescents with ID, addressing key questions about common assessment tools, their characteristics, and reported evidence. A systematic review was conducted of the literature published between 2010 and 2023 in the PsycINFO, ERIC, Education, and Psychology databases. An initial 1176 studies were reviewed by abstract, of which 485 were read in full text, leading to the selection and analysis of 22 studies. Most of the identified tools involve analyzing language samples obtained using wordless picture story books. Three common tools are emphasized. Studies have primarily identified inter-rater reliability and test-criterion evidence for validity. The main tools and their characteristics are discussed in depth to aid readers in discerning suitable options for research or practical applications. The importance of reporting diverse sources of evidence for validity and reliability within this population is highlighted.

## 1. Introduction

Oral narrative skill is the ability to produce and share a chronologically sequenced account of an event or story [1]. It involves both the overall organization and the inclusion of essential details (macrostructure) as well as the specific linguistic elements used within the story (microstructure) [2,3]. Thus, the act of producing narratives is both cognitively and linguistically demanding [4,5].

Children and adolescents with intellectual disabilities (ID) usually develop their oral narrative skills more slowly than typically developing (TD) children [6,7,8]. The significant limitations in intellectual functioning and adaptive behavior in individuals with ID [9] entail difficulties in functions and processes (i.e., language, cognition, executive functions, or working memory) that limit their narrative performance [8,10,11]. Therefore, it has been reported that individuals with ID generate less complex, cohesive, or coherent narratives than TD individuals [6,12]. Nevertheless, these skills are essential for different areas of development and relate to quality of life of people with ID (e.g., social inclusion, interpersonal relations) [13].

Findings on the narrative abilities of people with ID have been somewhat inconsistent [8]; this has been attributed to the diversity of tools or coding schemes used for assessment [10,14]. There are several methods for assessing narrative skills based on language samples, for example: scoring schemes such as the Narrative Assessment Protocol [15], Narrative Scoring Scheme [16], and Index of Narrative Complexity [17]; and tests such as the Bus Story Test [18] or the Narrative Competence Task [19]. However, tools originally created for use with TD children may pose difficulties if applied to individuals with ID without prior consideration or adaptation. Thus, awareness of the different modalities of assessment is crucial. For example, the generation of spontaneous stories requires skills (linguistic and cognitive) that may not be fully developed in individuals with ID at an early age [10], potentially resulting in a floor effect that could lead to inaccurate conclusions. Such challenges in the assessment of individuals with ID have been noted across different disciplines [20,21].

When interested in assessing narrative skills in children with ID it is necessary to be clear about the characteristics of the tools used, the type of narrative task required (generation/retelling, fictional/personal), the type of stimuli included, if it is a standardized tool or not, and the components assessed (i.e., the analysis scheme used). For instance, the type of task will be related to its utility for the assessment of certain elements. A story retelling task will present a different outcome to that of generating stories, because they evoke different components, and its usefulness will vary based on developmental stage [10,22]. Likewise, the type of stimuli used during the task can lead to disparate results [23]. In this regard, recognizing the characteristics of assessments and determining which ones to select and why are crucial steps in narrative assessment within this population.

On the other hand, it is important to know the psychometric properties that the different tools show in individuals with ID. The validity and reliability of an assessment are not static properties of a tool [24,25,26,27], and they need to be analyzed, especially when the tool is used for a population or context different from the one for which it was originally designed [27]. Both properties are variable, and various types of evidence can be provided. In this sense, it is important to explore the different sources of evidence of validity (e.g., content, internal structure, convergent, test criterion) or reliability (e.g., test–retest, internal structure, inter-rater) available for the tools [24].

While some studies have addressed the narrative skills in children and adolescents with ID of different etiologies (e.g., Down syndrome, fragile X syndrome, Williams syndrome) [23,28,29] there is no research that has delved into how to assess these skills in this population. To date, no study has synthesized and analyzed the tools used for assessing narrative skills in children and adolescents with ID. The aim of this study was to provide an overview of the tools for the assessment of oral narrative skills used with this population. This work considers aspects that have not been addressed in previous reviews [5,8,11,30,31]. For example, the recent review by Winters et al. [5] focused on the capability of the instruments to differentiate between children with TD and those with developmental language disorders, and excluded from their review studies with participants with ID. This review focuses on the assessments reported in recent research including different types of studies in which the narrative skills of children and adolescents with ID has been assessed. This work seeks to answer the following research questions:

- What are the most common tools to assess narrative skills in children and adolescents with ID?
- What are the characteristics of these tools, and which ones are most suitable for children and adolescents with ID?
- What is the evidence of reliability and validity of these assessment tools for this population?

## 2. Materials and Methods

A systematic review was conducted. According to PRISMA guidelines [32], a three-phase process was followed: (i) search strategy; (ii) selection and inclusion criteria; (iii) data extraction. The PRISMA checklist is presented in Table S1 (Supplementary Material). The review was not prospectively registered. The whole process of search and selection of studies is openly available in the Open Science Framework (OSF) (details in Data Availability Statement). Each followed phase is described in a separate section below.

### 2.1. Search Strategy

The search was limited to recent works published between January 2010 and December 2023 (inclusive of both dates). The language of articles was limited to English and Spanish. This notation pointed only to the language of the manuscript and not to the language of the participants of the studies (the selection of the studies allowed for any language spoken by the participants or any country). The search was carried out in four scientific databases, two specialized in psychology, PsycINFO (Ebsco, Ipswich, MA, USA) and Psychology Database (ProQuest, Ann Arbor, MI, USA), and two specialized in education, ERIC (Educational Resources Information Center) (Ebsco) and Education Database (ProQuest). Only articles published in peer-reviewed scientific journals were included. Search expanders or equivalent terms were not utilized. The search syntaxes were constructed considering the main terms used in the literature to refer to (i) narrative ability (ii) assessment, and (iii) the target population. For the search, only terms in English were used. The terms and syntax used for all databases were: “narrative skills” AND assessment AND children; “narrative language” AND measurement AND students; “narrative thinking” AND test AND children; “narrative abilities” AND tool AND children; “narrative abilities” AND evaluation AND children; “narrative competence OR narrative skills OR narrative abilities” AND children AND intellectual disabilit*. Given that the review is part of a larger review, most of the terms used did not limit the results only to studies with participants with ID. This criterion was applied later in the selection phase. Appendix A details the results obtained with each term and in each database as well as the composition of the initial pool of results after removing duplicates.

### 2.2. Selection and Inclusion Criteria

**Figure 1:**
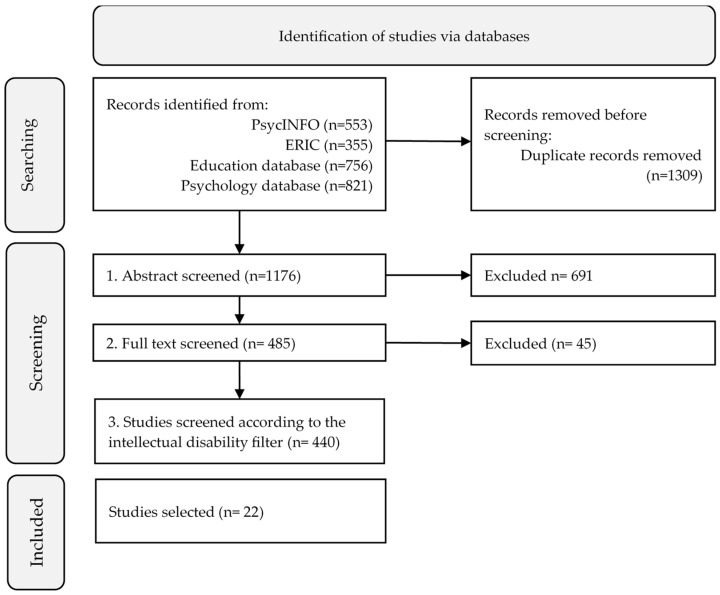
Flow diagram.

After identifying the initial pool of studies (n = 1176), the selection phase was undertaken. To be included, studies had to meet all the inclusion criteria. The inclusion criteria were addressed in a scaled manner in the selection of studies through the application of three consecutive filters (Figure 1) which allowed the final sample to be configured. The first filter (abstract level) verified the following criteria: (i) empirical studies; (ii) narrative skills assessed in children or adolescents; (iii) were not case studies; (iv) published between 2010 and 2023; and (v) manuscript written in English or Spanish. Empirical studies included descriptive studies, experiments, quasi-experimental studies, ex post facto designs, and instrumental studies. In a second filter (full text), the selection was limited to those studies in which (i) statistics on the measurements are included and (ii) oral narratives are assessed (e.g., written narratives were excluded). Finally, regarding the target population, all the studies that considered participants who were children or adolescents with ID were include, even if they also included adults, TD participants, or participants with other diagnosis (e.g., studies with participant with autism but without ID were excluded, participants with autism and ID were included). The final criterion applied was the inclusion of participants with ID (third filter). A total of 22 studies were selected for data extraction [2,6,7,22,28,29,33,34,35,36,37,38,39,40,41,42,43,44,45,46,47,48].

### 2.3. Data Extraction

The studies were analyzed and coded along two axes: (i) characteristics of the studies and (ii) characteristics of the assessments. The categories of analysis are detailed in Table 1 and Table 2. To categorize the type of study, the classification of quantitative empirical studies described by Montero and León [49] was used. The categories for the coding of characteristics of the assessments were delimited considering the categorization already used by Winters et al. [5]. The categories for coding validity and reliability included various types of evidence sources as outlined by AERA et al. [24]. (i.e., for reliability: internal consistency, test–retest, inter-rater; for validity: content validity, test criterion, convergent evidence, internal structure). However, only those found in the studies were reported. As will be detailed in the Results section, only some of these types of evidence were reported in the selected studies. This evidence was considered as reported, even if it was not explicitly or intentionally stated (e.g., study reports validity evidence referring to relationships with other linguistic variables theoretically related to narrative skills but does not report it as a validity outcome). The coding process was carried out by three judges who independently coded the selected studies. One judge coded all the selected studies, a second judge coded 82% (n = 18) of them, while a third judge coded 23% (n = 5). The three judges coded independently the categories analyzed. The reliability of the coding process was estimated using Krippendorff’s alpha [50], which indicated the degree of inter-rater agreement between the three judges for each of the categories. Perfect agreement (kalpha = 1.00) was obtained for the categories: % girls, % TD, ID etiology, IQ or MA, country, standardization, fictional or personal, type of reliability, and sources of validity, while an acceptable agreement (kalpha = 0.82 to 0.95) was obtained for the categories: study design, age, participants’ language, level of analysis, task type, and stimuli. All disagreements were resolved through discussion among the judges until consensus was reached for all categories. Following data extraction, the main results of the studies concerning the narrative skills of individuals with ID were summarized, although this is beyond the scope of the current paper. Table S2 (Supplementary Material) summarizes the main results of the selected studies in this regard.

## 3. Results

This section is organized into four subsections. First, the characteristics of the selected studies are described. Secondly, an analysis of the assessment tools identified in those studies is presented, highlighting their characteristics and the most used ones. Third, the reported reliability evidence of the tools is analyzed. Finally, the validity evidence of the tools available in the studies is examined.

### 3.1. Characteristics of the Selected Studies

Table 3 summarizes the characteristics of the studies (design and composition of the sample). Regarding their designs, they were mainly ex post facto and to a lesser extent quasi-experimental designs, corresponding to interventions [28,33]. The ex post facto studies were, to a greater extent, retrospective designs (n = 19) and, to a lesser extent, developmental designs (n = 1). Those of a retrospective type were conducted, in most cases (n = 13), with two comparison groups around a main measure (e.g., group with ID and TD group) (e.g., [34,35]) and in some cases (n = 6) by a single group evaluated with multiple measures (i.e., only participants with ID) (e.g., [6]). The sample size of the studies was generally small (M = 48; SD = 32.92; range = 8–129). The largest sample size (n = 129) was in the study of Estigarribia et al. [34].

Regarding the geographic location of the selected studies and the language of the participants, most of the studies had English-speaking participants (n = 14), mainly in the United States [2,6,29,34,36,37,38,39,40,41], but also in Canada [42], the United Kingdom [7,43], and New Zealand [44]. Four of the studies had Italian-speaking participants and were conducted in Italy [22,35,45,46]. Only two studies included Spanish-speaking participants [28,47], both from Spain. One of the studies considered Tamil-speaking participants in Sri Lanka [33], while another did not explicitly report the language but was conducted in Portugal [48].

As for the participants’ gender, the studies considered mixed samples in terms of gender, except for three studies that did not indicate the participants’ gender [35,42,45] and the work by Estigarribia et al. [34] that only included boys. Regarding the age, the variability was greater in studies that incorporated TD participants matched by MA with participants with ID. In these cases, very young TD children were included, whereas participants with ID were older (e.g., [22,29,34,36]). In any case, all studies included children or adolescents with ID, even if adults were also included [35]. In contrast, those studies that did not incorporate TD participants (n = 11) were more focused on a specific age range (e.g., [6,37,42]). Regarding the etiology of ID, in general, the studies included participants with Down syndrome (DS) (n = 13) [6,22,29,33,34,36,37,38,39,41,42,43,44], Williams syndrome (WS) (n = 6) [28,35,40,45,47,48], fragile X syndrome (FXS) (n = 6) [2,6,29,34,36,39], autism spectrum disorder (ASD) (n = 3) [33,34,39], and to a lesser extent, other diagnoses such as cerebral palsy (n = 1) [33], global development delay (GDD) (n = 1) [33], and Alexander disease (n = 1) [46] were included. Individuals with nonsyndromic ID were included in two studies [6,7]. Regarding the levels of ID, most of the studies reported middle to moderate ID according to reported IQ or MA of their participants (detailed in Table 3).

**Table 3:** Characteristics of the studies.

Study	Sample Size	Design Type/Study Design	% Girls	Age Range	% TD	Reported ID Etiology	Reported IQ or MA
Barton-Hulsey et al. (2017) [6]	n = 102	Ex post facto/retrospective with one group and multiple measures	45%	7;2–11;11	0%	DS, FXS, and nonsyndromic ID	IQ M (SD), range = 64.17 (10.64), 44–90
Brown et al. (2018) [7]	n = 109	Ex post facto/retrospective with two or more groups	40%	7;0–11;0 (ID) 4;0–11;0 (TD)	60%	Nonsyndromic ID	IQ range = 44–76; mild and moderate ID
Channell et al. (2015) [36]	n = 68	Ex post facto/retrospective with two or more groups	32%	10;3–15;6 (DS) 10;2–16;0 (FXS) 3;1–6;2 (TD)	34%	DS, FXS	Nonverbal IQ M (SD), range = 42.48 (70.7), 65–65 (DS); 44.41 (7.87), 36–65 (FXS)
Channell et al. (2020) [37]	n = 37	Ex post facto/retrospective with one group and multiple measures	65%	6;0–11;10	0%	DS	Nonverbal IQ M (SD), range = 59.92 (8.87), 41–75
Cleave et al. (2012) [42]	n = 32	Ex post facto/developmental longitudinal	NS	5;10–16;6	0%	DS	MA M, range = 4;9, 2;10–7;3
Diez-Itza et al. (2018) [28]	n = 8	Quasi experiment/pretest–post-test. One group	50%	8;11–24;4	0%	WS	IQ M, range = 64, 44–90
Estigarribia et al. (2011) [34]	n = 129	Ex post facto/retrospective with two or more groups	0%	6;2–15;10 (FXS) 6;3–15;11 (DS) 3;4–7;9 (TD)	30%	FXS, FXS-ASD, DS	MA M, range = 5;4, 3;4–7;8 (FXS); 5;2, 3;9–8;2 (DS)
Finestack et al. (2012) [29]	n = 57	Ex post facto/retrospective with two or more groups	42%	12;1–23;4 (DS) 11;4–19;9 (FXS) 3;7–7;9 (TD)	37%	DS, FXS	Nonverbal IQ M (SD), range = 41.71 (6.87), 36–57 (DS); 39.50 (6.05), 36–56 (FXS)
Gonçalves et al. (2011) [48]	n = 26	Ex post facto/retrospective with two or more groups	69%	11;0–29;0 (WS) 11;0–29;0 (TD)	50%	WS	IQ M (SD), range = 47.31 (7.05), 40–69
Hessling and Brimo (2019) [38]	n = 15	Ex post facto/retrospective with one group and multiple measures	67%	8;1–18;3	0%	DS	Nonverbal IQ M (SD) = 53.67 (14.17)
Hettiarachchi (2016) [33]	n = 30	Quasi experiment/pretest–post-test. One group	27%	3;2–15;0	0%	DS, CP, GDD, ASD	Mild and moderate ID. Neither IQ nor MA is reported.
Hogan-Brown et al. (2013) [39]	n = 94	Ex post facto/retrospective with two or more groups	0%	6;1–15; 0 (FXS) 6;7–15;1 (FXS–ASD) 4;2–12;9 (ASD) 6;10–14; 10 (DS) 3;0–8;0 (TD)	17%	FXS, FXS-ASD, ASD, DS	Nonverbal IQ M (SD), range = 58.89 (14.58), 38–89 (FXS); 54.04 (11.8), 40–79 (FXS–ASD); 69.65 (15.37), 40–102 (ASD); 53.12 (9.96), 38–73 (DS)
Jones (2013) [40]	n = 46	Ex post facto/retrospective with two or more groups	54%	8;0–14;5 (WS) 4;3–12;7 (TD)	61%	WS	Nonverbal IQ M (SD), range = 65.83(13.23), 41–84. MA range = 4;7–12;6
Laws and Hall (2014) [43]	n = 41	Ex post facto/retrospective with two or more groups	63%	3;9–11;1	0%	DS	IQ M (SD), range = 63.78 (14.46), 42–95.
Marini et al. (2010) [45]	n = 38	Ex post facto/retrospective with two groups	NS	6;0–25;0 (WS) 5;0–10;0 (TD)	76%	WS	IQ M (SD), range = 53.4 (6.7), 49–68. MA range = 5;0–10;1
Mastrogiuseppe and Lee (2017) [35]	n = 33	Ex post facto/retrospective with two or more groups	NS	8;5–39;0 (WS) 4;6–7;5 (TD)	67%	WS	MA range = 4;6–7;5. IQ not reported.
Michael et al. (2012) [41]	n = 18	Ex post facto/retrospective with two or more groups	56%	11;11–32;10 (DS) 3;2–13;6 (TD)	50%	DS	Only receptive vocabulary age is reported. Range = 3;2–13;6
Neal et al. (2022) [2]	n = 32	Ex post facto/retrospective with two or more groups	25%	12;5–18;0	0%	FXS	IQ M (SD), range = 37.67 (3.05), 36–48 (Males); 57.63 (18.64), 36–79
Pérez-García et al. (2015) [47]	n = 69	Ex post facto/retrospective with one group and multiple measures	42%	5;0–47;0	0%	WS	IQ M, range = 55.2, 40–96.
Van Bysterveldt and Guillon (2014) [44]	n = 25	Ex post facto/retrospective with one group and multiple measures	68%	5;11–13:1	0%	DS	Neither IQ nor MA is reported.
Zampini et al. (2023) [46]	n = 8	Ex post facto/retrospective with one group and multiple measures	50%	5;0–23;0	0%	Alexander disease	Neither IQ nor MA is reported.
Zanchi et al. (2021) [22]	n = 39	Ex post facto/retrospective with two or more groups	54%	10;7–15;2 (DS) 3;2–7;6 (TD)	67%	DS	MA range = 3;2–7;6.

NS = not specified; DS = Down syndrome; FXS = fragile X syndrome; NS-ID = nonsyndromic ID; WS = Williams syndrome; GDD = global development delay; ASD = autism spectrum disorder; CP = cerebral palsy; TD = typically developing. IQ = intelligence quotient. MA = mental age. M (SD) = mean (standard deviation).

### 3.2. Assessment Tools Identified and Their Characteristics

#### 3.2.1. Characteristics

Assessment tools were identified and coded based on their characteristics. Table 4 presents the characteristics analyzed. Each study and its elicitation procedure are reported in the first and second columns. In two of the selected studies, two different assessments were applied to assess narrative skills [33,42]. Therefore, although 22 studies were included, 24 assessment instances have been analyzed.

The first characteristic analyzed was whether the assessment was standardized or not (third column). Some of them correspond to language samples that use different types of stimuli with different scoring schemes (nonstandardized), while others correspond to language samples within standardized tests (which use a certain type of stimulus and scoring scheme and have defined interpretation norms). The assessment tools reported in the selected studies corresponded mostly to language samples without standardization and, to a lesser extent, to language samples within standardized tests (n = 4). However, of these four assessments within standardized test, two used the Bus Story test applying off-tool analysis schemes [34,42].

The second characteristic analyzed (fourth column) refers to the type of narrative task considered in the assessments: story generation or story retelling. The difference between the two is that in the retelling tasks, the examiner tells a story to the participants before asking them to retell the story in their own words; generation tasks are limited to delivering an instruction and/or a stimulus (e.g., illustrations) to elicit a story. For example, in the Narrative Competence Task (NCT), during the generation task, the examiner asks the child to browse the pages and then invites them to tell the story in their own words while browsing the pages again [22]. The tasks were mostly conducted in a modality of story generation (n = 17) and, to a lesser extent, story retelling (n = 7).

The third characteristic (fifth column) refers to the use of stimuli for elicitation. In most of the assessments, visual stimuli were used. In two assessments, audiovisual stimuli were used, which in both cases corresponded to wordless cartoon scenes. The stimuli used corresponded to illustrated and wordless storybook (n = 14) [2,6,22,29,34,36,37,38,39,42,43,46,47] (e.g., *Frog Goes To Dinner* story), illustrated and wordless plates or pictures used to convey or elicit stories (n = 4) [33,41,44,45] (e.g., illustrations of scenes, photographs), wordless cartoon scenes (n = 2) [28,35] (i.e., *Tom and Jerry* scenes), and illustrated storybooks with words (n = 2) [33,40] (e.g., *Peter and the Cat* story). Only two assessments were limited to verbal stimulus for the task (n = 2) [7,48], for instance, one study asked the participants to “Tell me a very important event in your life” [48]. In both cases, the use of solely verbal prompts corresponded to the elicitation of personal events.

The fourth characteristic analyzed (sixth column) corresponded to the nature of the stories used during the assessment. Most of them considered fictional stories, while only three were personal stories (e.g., accounts of personal experiences) [7,44,48].

**Table 4:** Characteristics of the assessments.

Study	Elicitation Procedure	Stand.	Task Type	Stimuli	Nature	Level of Analysis
Barton-Hulsey et al. (2017) [6]	Frog goes to dinner (FGTD) (story)	No	Generation	Illustrated and wordless storybook	Fictional	Macrostructure (includes ISL *) and microstructure
Brown et al. (2018) [7]	Account of a recent class-based event	No	Generation	Verbal prompts (oral)	Personal	Macrostructure (includes ISL)
Channel et al. (2015) [36]	FGTD/Frog on his own (FOHO) (stories)	No	Generation	Illustrated and wordless storybook	Fictional	Macrostructure (includes ISL) and microstructure
Channell et al. (2020) [37]	FGTD/FOHO (stories)	No	Generation	Illustrated and wordless storybook	Fictional	Microstructure and ISL
Cleave et al. (2012) [42]	Bus Story test (test)	Yes	Retelling	Illustrated and wordless storybook	Fictional	Macrostructure (includes ISL) and microstructure
	FOHO (story)	No	Generation	Illustrated and wordless storybook	Fictional	Macrostructure (includes ISL)
Diez-Itza et al. (2018) [28]	*Tom and Jerry* (cartoon)	No	Retelling	Cartoon scene (wordless)	Fictional	Macrostructure and microstructure
Estigarribia et al. (2011) [34]	Bus Story test (test)	Yes	Retelling	Illustrated and wordless storybook	Fictional	Macrostructure (includes ISL)
Finestack et al. (2012) [29]	FGTD (story)	No	Generation	Illustrated and wordless storybook	Fictional	Macrostructure (includes ISL) and microstructure
Gonçalves et al. (2011) [48]	Open-ended question about any personal significant life event	No	Generation	Verbal prompts (oral)	Personal	Macrostructure (includes ISL)
Hessling and Brimo (2019) [38]	FGTD (story)	No	Retelling	Illustrated and wordless storybook	Fictional	Macrostructure (includes ISL) and microstructure
Hettiarachchi (2016) [33]	*Peter and the Cat* (adapted story)	No	Retelling	Illustrated storybook	Fictional	Macrostructure and microstructure
	Saman and the baby elephant (story)	No	Generation	Illustrated and wordless plates or pictures	Fictional	Macrostructure and microstructure
Hogan-Brown et al. (2013) [39]	Adapted version of *Bed Full of Cats* (story)	No	Generation	Illustrated and wordless storybook	Fictional	Macrostructure (includes ISL) and microstructure
Jones (2013) [40]	Adapted version of *Thunder Cake* (story)	No	Generation	Illustrated storybook	Fictional	Microstructure
Laws and Hall (2014) [43]	*Frog, Where are You?* (FWAY) (story)	No	Generation	Illustrated and wordless storybook	Fictional	Microstructure
Marini et al. (2010) [45]	*Picnic and Cookie Theft* (two single pictures) and *Flowerpot and Quarrel* (two stories with six pictures each)	No	Generation	Illustrated and wordless plates or pictures	Fictional	Macrostructure and microstructure
Mastrogiuseppe and Lee (2017) [35]	*Tom and Jerry* (Cartoon)	No	Retelling	Cartoon scene (wordless)	Fictional	Microstructure
Michael et al. (2012) [41]	Own elaboration story	No	Retelling	Illustrated and wordless plates or pictures	Fictional	Microstructure
Neal et al. (2022) [2]	FGTD (story)	No	Generation	Illustrated and wordless storybook	Fictional	Macrostructure (includes ISL) and microstructure
Pérez-García et al. (2015) [47]	FWAY (story)	No	Generation	Illustrated and wordless storybook	Fictional	Macrostructure (includes ISL)
Van Bysterveldt and Guillon (2014) [44]	Photographs	No	Generation	Illustrated and wordless plates or pictures	Personal	Macrostructure and microstructure
Zampini et al. (2023) [46]	Narrative Competence Task (NCT) (test)	Yes	Generation	Illustrated and wordless storybook	Fictional	Macrostructure (includes ISL) and microstructure
Zanchi et al. (2021) [22]	NCT (test)	Yes	Generation	Illustrated and wordless storybook	Fictional	Macrostructure (includes ISL) and microstructure

* Stand. = standardized test; ISL = internal state language.

The fifth characteristic analyzed refers to the level of analysis of the narrative. The macrostructure refers to the organization of the story and the information provided [38]. Microstructure refers to quantifiable linguistic characteristics at the sentence and word level [3,6]. The internal state language (ISL) refers to the language used to provide information about the mental state of the characters of the story. In some scoring schemes, the ISL is categorized as one more component of the macrostructure, while some authors consider it an independent third level. The assessments reported in the selected studies were mainly mixed at the level analyzed. Mostly, they focused on the three levels of analysis, considering ISL as part of the macrostructure (n = 9) [2,7,22,29,36,38,39,42,46]. This was followed by assessments that focused on the macrostructure and microstructure, without considering the ISL (n = 5) [28,33,44,45]. Other assessments considered the macrostructure and ISL and did not consider the microstructure (n = 5) [7,34,42,47,48]. Some assessments (n = 4) focused only on the microstructure [35,40,41,43]. Only one assessment assessed microstructure together with the ISL [36].

The specific measures considered for each level are further detailed in Table 5. The macrostructural elements were assessed using different scoring schemes, such as the Narrative Scoring Scheme (NSS) (e.g., [29]) or the Story Grammar Scheme (e.g., [34]). As for the microstructural level, studies reported various types of microstructural measures for different purposes. For example, the mean length of the utterances (MLU) (in words or in morphemes) and the subordination indexes were reported as measures of syntactic complexity (e.g., [38]). The number of utterances or C-units and the number of total words (NTW or tokens) were reported as measures of productivity (e.g., [28]). As for measures of lexical diversity studies reported the number of different words (NDW) [6], type-token ratio (TTR) [41] or diversity index (D-index) [22].

#### 3.2.2. Most Common Tools

After analyzing the different assessments, it is worth highlighting those tools most frequently used in the population of interest, and their characteristics. The wordless picture book *Frog goes to Dinner* (FGTD) [51] was the story most commonly used as an elicitation procedure. It was used in both modalities of narrative task, as a generation task [2,6,29,36,37] and as a retelling task [38]. In all cases the tool was used with English-speaking participants. At a macrostructural level, the language samples elicited with the FGTD story were generally assessed using the Narrative Scoring Scheme [16,29], which considers specific elements of the story [2,6,29,37]. At a microstructural level, different measures were used (e.g., MLU, NDW). Other stories from the Frog series such as *Frog, Where are you?* (FWAY) and *Frog on his own* (FOHO) were also used. However, they were either employed as alternative stories to (FGTD) [36,37], analyzed with nonspecific macrostructural analysis schemes [42,47], or solely used for MLU analysis [43].

The second tool was the Bus Story test [18], that was used in two studies as a retelling task [34,42]. Although this test is embedded in a tool that corresponds to a standardized test with its own analysis scheme at the macrostructural level (Bus Story’s information score), the studies also analyzed the macrostructure using different analysis schemes independent of the test itself. These schemes include Story Grammar Schema and McKeough’s Story Structure Analysis. In both cases the tool was used with English-speaking participants.

The third tool is the NCT [19], which was used in two studies with Italian population with ID as a generation task [22,46]. This tool is a standardized test with its own analysis scheme for macrostructure, which considers the dimensions of events, structure, agents, anaphoric use of the article, and mental state lexicon. On the microstructural level, various measures were used (MLU, D-index, NTW, and subordinate clauses) [22,46].

### 3.3. Reliability Evidence Reported

Reliability is a key aspect of assessments and refers to the consistency of their scores [24]. To present evidence of reliability, the studies were analyzed based on the information available on the different sources. Although different types of evidence of reliability have been considered (as detailed in Table 2), the results are limited to those reported in the selected studies (Table 5). The studies can be classified as follows: (i) those that incorporate some reliability data for all measures of narrative skills conducted (n = 9) [7,29,34,35,37,38,39,40,48]; (ii) those that incorporate reliability data only for some of the measures of components assessed (n = 7) [2,6,22,36,42,44,45]; and (iii) those that do not report any reliability data (n = 6) [28,33,41,43,46,47]. Thus, of the 22 studies analyzed, 16 presented information regarding the reliability of their measures in at least one of the levels analyzed. Most of the studies that included reliability evidence did so only for the macrostructure measures, despite having analyzed microstructure aspects [2,6,22,36,42,44]. Some assessments included an analysis of the reliability of the microstructural measures (n = 6) [29,37,38,39,40,45].

As for the type of reliability evidence, studies only reported evidence of inter-rater reliability. On the one hand, this is understandable given that narrative analysis consists of making coding (microstructural) and scoring (macrostructural) decisions in which it is crucial to report evidence of agreement between two or more judges, to avoid coder bias. On the other hand, there is a lack of other types of evidence of reliability that could be valuable, such as test–retest reliability. As detailed in Table 5, the studies identified reported inter-rater reliability through different indices: (i) Krippendorff’s alpha (n = 4) [2,6,7,29]; (ii) percentage of agreement; (iii) Cohen’s kappa (n = 1) [34], or (iv) interclass correlation (n = 3) [34,39,48]. Most assessments only calculated the percentage of agreement (n = 8) [22,35,36,37,38,40,42,44]. It is important to notice that only some of these studies reported evidence exclusively considering participants with ID, as they did not include TD participants [2,6,37,38,42,44].

### 3.4. Validity Evidence Reported

Validity is the most fundamental property of assessments and refers to the degree to which evidence and theory support the interpretations of their use [24]. To present validity evidence reported, the studies were analyzed based on the information available on the different sources of validity. Although different types of evidence of validity have been considered (as detailed in Table 2), the results are limited to those reported (Table 5). As observed in Table 5, only some evidence of test-criterion relationships was identified. This is a kind of validity evidence based on relations to other variables and refers to the relation of the assessment (in this case of narrative skills) to a relevant criterion that is theoretically related to it [24]. The variable criteria were as follows: reading or literacy skills [6,38], vocabulary [2,37], emotion knowledge [37], receptive language [42], expressive language [42,43], memory skills [34,41], visual analysis abilities [45], and testimonial skills [7]. Some studies reported these correlations exclusively for individuals with ID [2,6,7,37,43]. These criterion variables were evaluated using standardized or systematic methods. In all cases, the evidence provided was not explicitly reported as evidence of validity, since the aim of the studies was not instrumental. Thus, these relationships were reported for other purposes (e.g., to predict).

Other types of evidence based on the relations with other variables such as convergent evidence, which corresponds to the extent to which one type of instrument correlates with another that measures the same thing (e.g., two tests that assess narrative skills), were not identified. In this regard, most studies used only one kind of tool to assess narrative skills, thus they could not report correlations. The studies that included more than one assessment [33,42] did not reported the correlation between them. For instance, Cleave et al. [42], who use two different tools (the Bus Story test and FOHO), mentioned that all macrostructural measures were highly correlated, but did not report those correlations.

## 4. Discussion

This work aimed to answer the following questions: (i) What are the most common tools to assess narrative skills in children and adolescents with ID?; (ii) What are the characteristics of these tools, and which ones are most suitable for children and adolescents with ID?; and (iii) What is the evidence of reliability and validity of these assessment tools for this population? These questions have already been partially answered in the results section. In this section some essential issues regarding these questions and the reported results are discussed. First, the three tools highlighted in the Results section are discussed according to their evidence and outcomes. Second, the characteristics of the tools and the suitability of each one for individuals with ID are discussed in depth. Third, the availability and importance of different sources of validity and reliability are discussed. Finally, limitations and projections of the work are stated.

### 4.1. What Are the Most Common Tools to Assess Narrative Skills in Children and Adolescents with ID?

The FGTD story, used for generation or retelling tasks and evaluated with the NSS, predominated in this population. Additionally, the Bus Story Test [18] and NCT [19] were commonly employed for retelling and generation tasks, respectively. Here, we briefly discuss the evidence of these tools for children and adolescents with ID. Additionally, we discuss the type of outcomes that these tools have provided in the literature for this population. Further details of the outcomes of each study can be consulted in the Supplementary Material (Table S2).

At macrostructural level, some studies reported inter-rater reliability evidence for FGTD story (using NSS) as a generation task [2,6,29] and as a retelling task [38]. At the microstructural level, different measures have been used (e.g., MLU), and some inter-rater reliability evidence has been reported as an indicator of process quality [29,38]. As for validity, some studies reported test-criterion evidence for their use at macrostructural level [6,38] (relation with reading skills), at microstructural level [2,38] (relation with reading skills and vocabulary) and at only ISL level [37] (relation with expressive vocabulary and emotion knowledge).

Regarding the type of results derived from its use, it can be observed that this tool (FGTD using NSS) has consistently yielded similar outcomes. For instance, various studies utilizing this tool have consistently reported strengths at the macrostructural level among individuals with ID, particularly in concerning the introduction of characters and settings (Introduction dimension of NSS) in comparison to other dimensions [2,6,29,36,38]. The performance in mental states has been less consistent. While some highlight the description of mental states as a strength [2] others report low performance [6], even in a retelling modality [38]. Furthermore, the results have been consistent in reporting similarities in macrostructural performance among different etiologies of ID (e.g., FXS, DS) [29,36]. Additionally, the tool has proven useful in identifying differences between etiologies in certain components [29], as well as in distinguishing their performance from TD groups matched by MA or MLU [2,29,36]. This tool has been also used to identify variables related to narrative performance (such as MA or literacy skills) (e.g., [6,37,38]), and to explore difference by gender within etiologies (e.g., female with FXS and male with FXS) [2]. As for microstructure (outside the NSS), the outcomes have been consistent in showing restricted performance [29,38].

The use of this tool has not only revealed limitations but also strengths. This suggests that the tool may not exhibit a floor effect (nor a ceiling effect), making it suitable for the ID population. Accordingly, authors such as Finestack et al. [29] explicitly conclude that NSS (applied to the FGTD) may serve as a valuable tool for individuals with ID. Thus, despite some suggestions [10] that spontaneous story generation tasks may encounter floor effects in this population, using this tool in a generation mode, with the support of visual elicitation stimuli (nonspontaneous) (storybook) and analyzed with the NSS, may alleviate this concern.

As for the Bus Story test [18], used in two studies as a retelling task [34,42], some evidence can be highlighted. At a macrostructural level, Estigarribia et al. [34] reported inter-rater reliability for the scoring of this story using the Story Grammar Schema. The same author reported some test-criterion evidence (relationship between macrostructure in a retelling task and short-term memory). Cleave et al. [42] reported inter-rater reliability for the scoring of the macrostructure of the story using the McKeough’s Story Structure Analysis scheme and reported the test-criterion correlation between macrostructure and receptive language. At microstructural level Cleave et al. [42] reported a test-criterion correlation between MLU and both receptive and expressive language.

Regarding the type of results derived from its use, the Bus Story Test [18] has been used in longitudinal studies to assess narrative skill development in individuals with ID [42] Additionally, the Bus Story Test has been employed to compare narrative skills across different etiologies of ID, such as FXS and DS [34]. The study found similar macrostructural performance, consistent with outcomes obtained through other methods, but also identified differences between specific diagnoses, such as FXS and FXS-ASD. This tool has also been used to identify related variables with narrative performance [34]. Since the tool has proven useful in identifying changes over time or differences between ID groups, it likely does not exhibit a floor effect in this population. In comparison to the frog story FOHO, the Bus Story Test was found to generate longer narratives by Cleave et al. [42]. This finding may be relevant for practitioners or researchers seeking to elicit longer narratives. However, this difference may be attributed to the use of generation mode for the FOHO story and retelling mode for the Bus Story Test.

Finally, in relation to the NCT [19], some points are noteworthy. The study by Zanchi et al. [22] provided some evidence of inter-rater reliability at the macrostructural level for this tool (as an indicator of process quality). While there is limited psychometric evidence of its performance specifically in individuals with ID, it remains the only standardized tool for assessing narrative skills with interpretation norms available for the Italian population (TD). The NCT tool has been useful in reporting outcomes in Italian-speaking children with ID, with results consistent with those obtained using other tools in different languages. For example, similar macrostructural performance was reported between children with ID and TD children matched by MA [46]. Additionally, the NCT has been used to explore narrative skills in Italian population with different etiologies of ID (i.e., Alexander disease and DS) [46] as well as to compare the performance of Italian children with ID and TD children matched by different criteria (i.e., MA, MLU) [22]. Notably, the NCT employs a very simple and colorful story depicting a familiar situation (children in a park) [19], in contrast to FGTD, which presents a story in black and white set in a less familiar context (“fancy restaurant”). In this regard, Zanchi et al. [22] emphasize that the simplicity of the NCT makes it suitable for use with young children and children with ID.

### 4.2. What Are the Characteristics of These Tools, and Which Ones Are Most Suitable?

In this section, some aspects and implications associated with these characteristics are discussed to help the reader (research or practitioner) reflect on the suitability of the different assessment modalities.

All the assessments that have been applied to assess narrative skills in children and adolescents with ID in recent years correspond to analysis of language samples. Most of them were not part of standardized tests and utilized different types of stimuli with different scoring schemes. This has some important implications. On the one hand, this result is related to the flexibility of this type of instrument compared to language samples within standardized tests. In fact, in some cases, this type of instrument was used due to the lack of standardized tools adapted to a certain context. For example, the study by Hettiarachchi et al. [33] was carried out in the Tamil language. Likewise, language samples can be particularly useful in populations in which the conditions of application of standardized tools tend to have a floor effect [52]; therefore, they may be preferable in populations with ID.

On the other hand, the use of nonstandardized tools based on language samples instead of language samples tasks within standardized tests has some disadvantages. One of them is that it makes it difficult to compare the results between studies, replicate the assessment conditions as well as to evaluate their psychometric quality. However, this can be remedied by using application protocols for narrative tasks and predefined scoring schemes. For example, in SALT Software website, [53] Mayer’s Frog stories have been accompanied by a series of materials to schematize their use. In this way, scripts have been developed to standardize the way storybooks are presented (e.g., indicating what the examiner should say on each sheet). Likewise, that team provided a NSS for each story. Initiatives such as this provide greater control to the assessment conditions through nonstandardized narrative tasks, and with this, greater internal validity to the conditions of the studies conducted as well as greater replicability and comparability between different studies.

Regarding the task type (modality), narrative tasks in the mode of story generation were more frequent in the literature than the mode of retelling. This has relevant repercussions on the population of children and adolescents with ID because both modalities differ in the amount of information provided for the task as well as in the memory demands involved in each modality. While the retelling tasks provide the examinee with a structure of the story, in the generation tasks, the intervention of the examiner is limited to delivering an instruction and/or a stimulus, so that the structure of the story depends to a greater extent on the examinee. On the other hand, while the retelling task demands long-term memory, the generation task will demand more working memory [12,54]. In any case, the decision of one type of task or another will be relevant because both modalities evoke different components of the narrative, and its usefulness will vary according to the age of those evaluated [10].

Although most of the studies analyzed used the narrative generation modality, the retelling modality has been advocated for use in children with ID because it allows greater narrative production than does a generation task [34]. Likewise, although they have been considered to be effective as generation tasks, retelling tasks have been noted for providing longer stories with more grammatical components of the story, requiring less time to transcribe, and providing more reliable scores [55]. Furthermore, the value of retelling tasks has been supported to evaluate the comprehension of stories [56].

Regarding the use of elicitation stimuli, most of the assessments considered the use of stimuli in narrative tasks. The stimuli chosen were mostly images (i.e., wordless picture books, pictures, and wordless illustrations that were not books), although audiovisual records were also used (i.e., wordless cartoon scene). This is relevant because the type of narrative task and the use of supportive stimuli influence the narratives produced [23,55]. For instance, wordless picture-story books or picture-story sequences involve comprehension skills and this has implications in assessment [57,58]. Although the use of images as a supportive stimulus to elicit narratives could limit the type and amount of information produced, their use is recommended to facilitate the task in children and/or adolescents with ID because it reduces processing difficulties [55]. In this regard, it has been recommended that the stimuli remain available during the narrative task (not only in the instructions or presentation of the story) and thus lower the demand on working memory and facilitate a greater narrative repertoire. In fact, Cleave et al. [42] emphasize that visual support is essential for individuals with ID to demonstrate their abilities to the fullest. For their part, audiovisual stimuli, which are more innovative in the literature, have been highlighted for their value in facilitating the understanding of the narrative structure [28].

With regard to the nature of the stories used, in most of the studies analyzed, the authors opted more for the use of fictitious stories than for personal accounts. This decision is not accidental because it has relevant implications for the elicitation of stories with children in general and with children with ID. Fictional stories provide a built-in story structure that alleviates the cognitive load of the narrative (compared to a personal narrative) [37]. Likewise, if the focus of interest is on the production of ISL, the use of fictional stories can be advantageous. Channell et al. [37] indicates that because fictional stories focus on other characters (not the self), they provide an optimal context to provoke the use of language of mental states.

### 4.3. What Is the Evidence of Reliability and Validity of These Assessment Tools for This Population?

Some relevant ideas can be drawn from this work in relation to the psychometric properties of the assessments. First, it is possible and important to report reliability and validity evidence of assessment, even if the instrument used is not standardized. In this context, nonstandardized assessments based in language samples analysis can and should consider some reliability and validity evidence.

Of the studies analyzed, several of them did not report any validity or reliability evidence in their assessments. This is understandable given the studies were not on instrumental focus, this was not their aim. Thus, the lack of such evidence is not a criticism of the quality of these studies, but rather reflects a gap in the literature on this aspect. The assessment tools used may have shown evidence of reliability and validity previously -for example in standardization studies (e.g., [19])—but not in populations with ID. No instrumental studies were identified that focused on providing evidence of psychometric properties of any instrument in this specific population.

Of the evidence that was identified, other sources of evidence of reliability and validity would be desirable. On reliability, only inter-rater reliability evidence was identified. In the studies this type of evidence is reported as an indicator of the quality of the transcription and coding process of language samples. In this sense, it refers to the measurement error coming from the coder. This is important since language analysis is influenced by the coder’s decisions from transcription (e.g., segmentation of utterances) to the scoring of macrostructural aspects. However, there is a lack of other types of reliability evidence that are crucial as indicators of the consistency of assessments, such as evidence of test–retest reliability (consistency over time) or internal consistency. Each kind of reliability evidence should not be considered equivalent, as each includes a unique definition of measurement error [24]. On validity, some evidence, specifically test-criterion evidence, has been reported for some of the assessments. There is a lack of other sources of validity evidence that are fundamental and may vary among specific groups [24], such as convergent evidence (correlations between different tools that assess narrative abilities) or internal structure evidence (degree to which the empirical grouping of the different elements occurs in a coherent way with the sub-dimensions considered in the tool). Providing this kind of evidence could offer insights into their measurement validity and into their differential performance in specific situations.

Addressing the need for more diverse evidence on the reliability and validity of assessment tools of the narrative skills in children and adolescents with ID requires future empirical studies focused on their application and analysis in this population.

### 4.4. Study Limitations and Projections

In this study, an updated and rigorous review focused on the assessment of narrative skills in a specific group has been conducted. However, this study is subject to limitations inherent in a systematic review. The studies selected and analyzed are the result of a certain choice of databases, search terms, language, and periods of time that could leave out relevant works. Another limitation of a systematic review is that the aspects coded and analyzed may exclude other relevant elements. For example, the use of paraverbal elements during the narrative tasks (such as gestures or the prosody of the story) that were considered in some studies [22,33,35], were not coded. An aspect overlooked is the nature of the characters in the narrative, such as whether they are human characters (e.g., *Thunder Cake*), animals (e.g., *Frog Where Are You?*), or originally inanimate objects (e.g., the Bus Story Test) are considered. Although stories that include animals are frequent and are recommended for young children some authors consider it preferable that stories include human characters. In this sense, it has been mentioned that the appearance of human characters favors disambiguation and the use of pronouns and that characters carrying out realistic activities familiar to the child favors understanding [40], while unknown experiences hinder understanding [33]. In the same sense, it has been indicated that the use of more realistic characters facilitates the task in children with ID [33].

The main outcomes of each study on narrative skills of individuals with ID (summarized in Table S2) have been discussed in relation to the most common assessments. However, future studies should delve deeper into analyzing the outcomes obtained for this population using different types of assessment. This way, possible discrepancies obtained by the assessment methods could be further investigated.

Another projection pertains to the observed differences among groups of individuals with ID, including factors like chronological age (CA), gender, and language. When it comes to CA, no significant trends were found when comparing children and adolescents with ID in the reviewed studies. CA did not appear to be a relevant factor when contrasted with MA or literacy skills. In Neal’s study [2], encompassing adolescents with FXS, CA did not predict narrative performance significantly. Nevertheless, some studies suggest that CA is relevant, as developmental changes in narrative content have been observed from the youngest to the oldest participants with DS (two years to eight years) [59]. Regarding gender, certain studies, like Neal et al. [2] identified differences between boys and girls with FXS, indicating significant disparities in narrative performance. Conversely, Mastrogiuseppe and Lee [35] found no gender-based distinctions in narrative performance among individuals with WS. As for language, no discrepancies were reported across different languages, as no study involving participants with ID included more than one spoken language. Future research could further explore developmental differences among children and adolescents with ID, as well as differences related to gender or language, while also considering the impact of different types of assessments on these findings.

This work addresses various aspects that may concern researchers or practitioners interested in evaluating narrative skills in children or adolescents with ID. It provides insights into the most common tools, their characteristics, and the evidence supporting their use, as well as the types of outcomes they can provide. With this information, stakeholders can make informed decisions about the most appropriate tools for their specific purposes. For example, a researcher may want to explore the oral narrative skills of children with ID and aim for long narratives. In such cases, a retelling task using visual supports, such as the Bus Story test or FGTD in retelling mode, may be optimal. Conversely, a practitioner may be interested in assessing the narrative production skills in very young children with ID, without particular focus on narrative length. In this scenario, may be most suitable to use a generation task with simple visual stimuli, such as the NCT. We hope that these and other questions can be answered in the work presented here.

## Figures and Tables

**Table 1:** Categories of analysis for the characteristics of the studies.

Criteria	Options or Description	Subtypes or Description
Sample size	Total simple size	
Study design *	Descriptive	Systematic observation (naturalistic observation; structured observation)/descriptive study of populations through survey research (cross-sectional, longitudinal)
	Experiments	With different groups (between subjects)/with the same group (within subjects)/factorial experiments
	Quasi experiments	Pretest–post-test/post-test only/interrupted time series
	Ex post facto	Retrospective/prospective/developmental (transversal, longitudinal, sequential)
	Single-subject experiments	No-reversal design/reversal design/design with treatments (two different treatments; levels of treatments; interaction of two treatments)/multiple baseline
	Instrumental	Instrumental design
% Girls	Percentage of girls included	
Age	Age range (Y; M–Y; M)	
% TD	Percentage of TD participants	
ID Etiology	Etiology of the ID	
IQ or MA	Reported IQ or mental age (MA) of participants with ID	
Country	e.g., United States, Spain, Italy	
Participants’ language	e.g., English, Spanish, Italian	

* Taxonomy of empirical quantitative studies from Montero and León [49].

**Table 2:** Categories of analysis according to the characteristics of the assessment conducted.

Criteria	Categories and Examples
Standardized test	Yes (i.e., language samples that use a certain type of stimulus and scoring scheme. It has norms of interpretation.)/no (i.e., language samples that use diverse types of stimuli with different scoring schemes. It has no norms of interpretation).
Task type	Story generation/story retelling
Fictional or personal	Fictional (e.g., tale)/personal (e.g., autobiographical stories)
Stimuli	Illustrated and wordless storybook (e.g., frog goes to dinner)/illustrated and wordless plates or pictures (e.g., images, pictures, draws)/cartoon scene (wordless) (e.g., *Tom and Jerry*)/verbal prompts (oral) (e.g., instruction, prompts, questions)/illustrated story book with words (e.g., *Peter and the Cat*).
Level of analysis	Macrostructure (e.g., story grammar); microstructure (e.g., lexical diversity); internal state language (ISL) (e.g., emotions); mixed (e.g., macrostructure and microstructure)
Type of reliability evidence and reliability data available *	e.g., inter-rater reliability (e.g., Krippendorff’s alpha, Cohen’s kappa), internal consistence (e.g., ordinal alpha, Cronbach’s alpha), test–retest (e.g., Pearson correlation coefficient)
Sources of validity evidence and validity data available *	e.g., Evidence based on test content, internal structure, on relations to other variables (convergent, discriminant, test-criterion relationships).

* For both validity and reliability, different types of evidence sources contemplated by AERA et al. [24] were considered.

**Table 5:** Components or measures analyzed and available reliability and validity evidence.

Study	Components Analyzed	Reliability Data Available	Validity Data Available
Barton-Hulsey et al. (2017) [6]	Macrostructure (includes ISL). Narrative Scoring Scheme (NSS): introduction, character, mental state, referencing, conflict, cohesion, and conclusion.	Inter-rater reliability. Krippendorff’s alpha for each dimension	Test-criterion evidence. Correlations between NSS and reading skills.
	Microstructure: MLUm, NDW, total utterances, % intelligible.	-	Test-criterion evidence. Moderate correlations between microstructure (MLU and NDW) and reading skills.
Brown et al. (2018) [7]	Macrostructure (includes ISL). Story grammar elements: initiating event, internal response, plan, attempt, outcome, and reaction (emotion or actions).	Inter-rater reliability. Krippendorff’s alpha for total score and range for dimensions.	Test criterion evidence. Correlations between macrostructure and testimonial skills (only participants with ID).
Channell et al. (2015) [36]	Macrostructure (includes ISL). Story grammar elements.	Inter-rater reliability. Percent agreement for each dimension.	-
	Microstructure. MLU, verb use, adverb use, conjunction use.	-	-
Channell et al. (2020) [37]	Microstructure. MLU	Inter-rater reliability. Percent agreement.	-
	Internal state language (ISL): mental state language (MSL) density, MSL diversity	Inter-rater reliability. Percent agreement.	Test-criterion evidence. Correlations between mental state language and expressive vocabulary knowledge and emotion knowledge.
Cleave et al. (2012) [42]	Macrostructure (includes ISL) (Bus Story). McKeough’s Story Structure analysis and Bus Story’s information score.	Inter-rater reliability. Percent agreement for story structure.	Test-criterion evidence. Correlations between macrostructure and receptive language.
	Microstructure (Bus Story): MLU in five longest T-units, average clauses per T-unit, NTW, NDW.	-	Test-criterion evidence. Correlations between microstructure and receptive and expressive language.
	Macrostructure (includes ISL) (FOHO). McKeough’s Story Structure analysis	Inter-rater reliability. Percent agreement for story structure.	-
Diez-Itza et al. (2018) [28]	Macrostructure. PRE-CORP: scenarios, episodes, events, characters. Productivity (completeness) and complexity (sequential order)	-	-
	Microstructure. Productivity: utterances, clauses, tokens; complexity: syntactic complexity, lexical diversity; cohesion: MLU, types, markers.	-	-
Estigarribia et al. (2011) [34]	Macrostructure: story grammar schema	Inter-rater reliability. Interclass correlation for total score and Cohen’s kappa for each dimension.	Test-criterion evidence. Correlations between macrostructure of a story retelling and short-term memory.
Finestack et al. (2012) [29]	Macrostructure (includes ISL): NSS (using an adapted rubric of its own).	Inter-rater reliability. Krippendorff’s alpha for each dimension.	-
	Microstructure: C-Units, MLU	Inter-rater reliability. Percent agreement for both measures.	-
Gonçalves et al. (2011) [48]	Macrostructure (includes ISL): System for the Assessment of the Structural Coherence of Narrative/System for the Assessment of Narrative Content Diversity/System for the Assessment of Narrative Process Complexity.	Inter-rater reliability. Interclass correlation for all sub dimensions.	-
Hessling and Brimo (2019) [38]	Macrostructure (includes ISL): NSS	Inter-rater reliability. Percent agreement for total.	Test-criterion evidence. Correlations between macrostructure (NSS) and literacy skills.
	Microstructure: MLU, NDW, Narrative Assessment Protocol (NAP).	Inter-rater reliability. Percent agreement for total NAP.	Test-criterion evidence. Correlations between microstructure (MLUm, NDW, NAP) and literacy skills.
Hettiarachchi (2016) [33]	Macrostructure (both stories): content (information score) based on the production of key aspects of the story	-	-
	Microstructure (both stories): MLU, C-units, syntactic structures	-	-
Hogan-Brown et al. (2013) [39]	Macrostructure (includes ISL): Evaluative Coding Scheme, story structure (main episodes) and thematic maintenance (theme and resolution)	Inter-rater reliability. Interclass correlation for all measures.	-
	Microstructure: MLUm, number of clauses, complex syntax and its diversity.	Inter-rater reliability. Percent agreement for MLUm	-
Jones (2013) [40]	Microstructure: grammatical errors, referential cohesion errors, tense shifting errors, connective cohesion errors.	Inter-rater reliability. Percent agreement for three of four dimensions.	-
Laws and Hall (2014) [43]	Microstructure: MLU	-	Test-criterion evidence. Correlations between MLU and expressive language.
Marini et al. (2010) [45]	Macrostructure: informative content (lexical informativeness, % thematic informativeness), discursive organization (% local coherence errors, % global coherence errors).	-	Test-criterion evidence. Correlations between macrostructural measures and visual analysis abilities.
	Microstructure: productivity: NDW, speech rate NDW/min. Lexical processing: NDW/n units, number of semantic paraphasias, % parapragmatic errorsMorphosyntactic organization: MLU, % complete sentences	Inter-rater reliability. Percent agreement for all measures.	Test-criterion evidence. Correlations between microstructural measures and neuropsychological scores were assessed but no significant correlation was reported.
Mastrogiuseppe and Lee (2017) [35]	Microstructure: MLU, number of clauses, use of spatial language (e.g., verbs).	Inter-rater reliability. Percent agreement for some measures.	-
Micheal et al. (2012) [41]	Microstructure: MLU, NTW, % utterances, TTR, target verb produced, verbs produced with correct argument structure.	-	Test-criterion evidence. Correlations between microstructural performance and memory skills.
Neal et al. (2022) [2]	Macrostructure (includes ISL): NSS, using rubric of Finestack (2012) and own adaptions	Inter-rater reliability. Krippendorff’s alpha for each dimension.	Test-criterion evidence. Correlations between macrostructure (NSS) and vocabulary (expressive and receptive) and literacy skills (written language).
	Microstructure: MLUm	-	Test-criterion evidence. Correlations between microstructure measures (MLUm) and vocabulary (expressive and receptive).
Pérez-García et al. (2015) [47]	Macrostructure (includes ISL): scoring system modified by the author.	-	-
Van Bysterveldt and Guillon (2014) [44]	Macrostructure: personal narrative quality (PNQ).	Inter-rater reliability. Percent agreement for total score.	-
	Microstructure: MLUm, NDW	-	-
Zampini et al. (2023) [46]	Macrostructure (includes ISL): events, structure, agents, anaphoric use of the article, mental state lexicon	-	-
	Microstructure: NTW, MLU, subordinate clauses (implicit and explicit)	-	-
Zanchi et al. (2021) [22]	Macrostructure (includes ISL): events + agents, structure.	Inter-rater reliability. Percent agreement for both elements.	-
	Microstructure: NTW, D index, MLU, syntactic complexity	-	-

(-): The hyphen indicates that this type of information was not reported; MLU: mean length of utterance in words; MLUm: mean length of utterance in morphemes; NDW (Number of different words); Total words (NTW); PRE-CORP: Pragmatic Evaluation Protocol for the analysis of oral Corpora.

## Data Availability

The data detailing the study selection process are publicly available in the Open Science Framework: https://osf.io/tg56j/?view_only=578c95def1bc485ca87fddc802708dba (accessed on 25 February 2024).

## Supplementary Materials

The following supporting information can be downloaded at: https://www.mdpi.com/article/10.3390/bs14040308/s1, Table S1: PRISMA checklist. Table S2. Main results of studies on narrative skills of individuals with ID.

## Appendix A

**Table A1:** Retrieval results and removal of duplicate records.

Terms	Database	Records	Total	Duplicate Removed	Selected for Screening
“narrative skills” AND assessment AND children	PsycINFO	263	2485	1309	1176
	ERIC	112			
	Education database	409			
	Psychology database	404			
“narrative language” AND measurement AND students	PsycINFO	46			
	ERIC	154			
	Education database	5			
	Psychology database	141			
“narrative thinking” AND test AND children	PsycINFO	22			
	ERIC	15			
	Education database	17			
	Psychology database	17			
“narrative abilities” AND tool AND children	PsycINFO	62			
	ERIC	31			
	Education database	156			
	Psychology database	140			
“narrative abilities” AND evaluation AND children	PsycINFO	142			
	ERIC	43			
	Education database	166			
	Psychology database	112			
“narrative competence OR narrative skills OR narrative abilities” AND children AND intellectual disabilit*	PsycINFO	18			
	ERIC	0			
	Education database	3			
	Psychology database	7			

(*) Include all words with the root “disabilit-”(such as disability or disabilities).

## References

[1] Bowles R.P., Justice L.M., Khan K.S., Piasta S.B., Skibbe L.E., Foster T.D. (2020). Development of the Narrative Assessment Protocol-2: A tool for examining young children’s narrative skill. Lang. Speech Hear. Serv. Sch..

[2] Neal C.N., Brady N.C., Fleming K.K. (2022). Narrative analysis in adolescents with fragile X syndrome. Am. J. Intellect. Dev. Disabil..

[3] Petersen D.B., Gillam S.L., Spencer T., Gillam R.B. (2010). The effects of literate narrative intervention on children with neurologically based language impairments: An early stage study. J. Speech Lang. Hear. Res..

[4] Botting N. (2002). Narrative as a tool for the assessment of linguistic and pragmatic impairments. Child Lang. Teach. Ther..

[5] Winters K.L., Jasso J., Pustejovsky J.E., Byrd C.T. (2022). Investigating narrative performance in children with developmental language disorder: A systematic review and meta-analysis. J. Speech Lang. Hear. Res.

[6] Barton-Hulsey A., Sevcik R.A., Romski M. (2017). Narrative language and reading comprehension in students with mild intellectual disabilities. Am. J. Intellect. Dev. Disabil..

[7] Brown D.A., Brown E., Lewis C.N., Lamb M.E. (2018). Narrative skill and testimonial accuracy in typically developing children and those with intellectual disabilities. Appl. Cogn. Psychol..

[8] Segal A., Pesco D. (2015). Narrative skills of youth with Down syndrome: A Comprehensive literature review. J. Dev. Phys. Disabil..

[9] Schalock R.L., Luckasson R., Tassé M. (2021). Intellectual Disability: Definition, Diagnosis, Classification, and Systems of Supports.

[10] Blom E., Boerma T. (2016). Why do children with language impairment have difficulties with narrative macrostructure?. Res. Dev. Disabil..

[11] Favot K., Carter M., Stephenson J. (2021). The effects of oral narrative intervention on the narratives of children with language disorder: A Systematic Literature Review. J. Dev. Phys. Disabil..

[12] Baixauli-Fortea I., Miranda Casas A., Berenguer-Forner C., Colomer-Diago C., Roselló-Miranda B. (2019). Pragmatic competence of children with autism spectrum disorder. Impact of theory of mind, verbal working memory, ADHD symptoms, and structural language. Appl. Neuropsychol. Child.

[13] Schalock R.L., Verdugo M.A. (2002). Handbook on Quality of Life for Human Service Practitioners.

[14] Peterson C. (2011). Children’s memory reports over time: Getting both better and worse. J. Exp. Child Psychol..

[15] Justice L.M., Bowles R., Pence K., Gosse C. (2010). A scalable tool for assessing children’s language abilities within a narrative context: The NAP (Narrative Assessment Protocol). Early Child. Res. Q..

[16] Heilmann J., Miller J.F., Nockerts A., Dunaway C. (2010). Properties of the Narrative Scoring Scheme Using Narrative Retells in Young School-Age Children. Am. J. Speech Lang. Pathol..

[17] Petersen D.B., Gillam S.L., Gillam R.B. (2008). Emerging procedures in narrative assessment: The Index of Narrative Complexity. Top. Lang. Disord..

[18] Renfrew C. (1991). The Bus Story.

[19] Zanchi P., Zampini L. (2020). The Narrative Competence Task. Eur. J. Psychol. Assess..

[20] DiStefano C., Sadhwani A., Wheeler A.C. (2020). Comprehensive assessment of individuals with significant levels of intellectual disability: Challenges, strategies, and future directions. AJIDD-Am. J. Intellect. Dev. Disabil..

[21] Ludi E., Ballard E.D., Greenbaum R., Pao M., Bridge J., Reynolds W., Horowitz L. (2012). Suicide risk in youth with intellectual disabilities: The challenges of screening. J. Dev. Behav. Pediatr..

[22] Zanchi P., Zampini L., Panzeri F. (2021). Narrative and prosodic skills in children and adolescents with Down syndrome and typically developing children. Int. J. Speech Lang. Pathol..

[23] Miles S., Chapman R., Sindberg H. (2006). Sampling context affects MLU in the language of adolescents with Down syndrome. J. Speech Lang. Hear. Res..

[24] American Educational Research Association (AERA), American Psychological Association (APA), National Council on Measurement in Education (NCME) (2014). Standards for Educational and Psychological Testing.

[25] Cizek G. (2020). Validity: An Integrated Approach to Test Score Meaning and Use.

[26] Cook D., Brydges R., Ginsburg S., Hatala R. (2015). A contemporary approach to validity arguments: A practical guide to Kane’s framework. Med. Educ..

[27] International Test Commission (ITC) (2017). The ITC Guidelines for Translating and Adapting Test.

[28] Diez-Itza E., Martínez V., Pérez V., Fernández-Urquiza M. (2018). Explicit oral narrative intervention for students with Williams syndrome. Front. Psychol..

[29] Finestack L.H., Palmer M., Abbeduto L. (2012). Macrostructural narrative language of adolescents and young adults with Down syndrome or fragile X syndrome. Am. J. Speech Lang. Pathol..

[30] Petersen D.B. (2011). A systematic review of narrative-based language intervention with children who have language impairment. Commun. Disord. Q..

[31] Portilla A.Y., Almanza V., Castillo A.D., Restrepo G. (2021). El desarrollo de las habilidades narrativas en niños: Una revisión sistemática de la literatura. Rev. Investig. Logop..

[32] Page M.J., McKenzie J.E., Bossuyt P.M., Boutron I., Hoffmann T.C., Mulrow C.D., Shamseer L., Tetzlaff J.M., Akl E.A., Brennan S.E. (2021). The PRISMA 2020 statement: An updated guideline for reporting systematic reviews. BMJ.

[33] Hettiarachchi S. (2016). The effectiveness of Colourful Semantics on narrative skills in children with intellectual disabilities in Sri Lanka. J. Intellect. Disabil..

[34] Estigarribia B., Martin G.E., Roberts J.E., Spencer A., Gucwa A., Sideris J. (2011). Narrative skill in boys with fragile X syndrome with and without autism spectrum disorder. Appl. Psycholinguist..

[35] Mastrogiuseppe M., Lee S.A. (2017). What gestures reveal about cognitive deficits in Williams Syndrome. Dev. Neuropsychol..

[36] Channell M.M. (2015). Narrative language competence in children and adolescents with Down syndrome. Front. Behav. Neurosci..

[37] Channell M.M. (2020). Cross-sectional trajectories of mental state language development in children with Down syndrome. Am. J. Speech Lang. Pathol..

[38] Hessling A., Brimo D.M. (2019). Spoken fictional narrative and literacy skills of children with Down syndrome. J. Commun. Disord..

[39] Hogan-Brown A.L., Losh M., Martin G.E., Mueffelmann D.J. (2013). An investigation of narrative ability in boys with autism and Fragile X syndrome. AJIDD Am. J. Intellect. Dev. Disabil..

[40] Jones N.E. (2013). The use of cohesive markers in narratives by children with Williams syndrome. Appl. Psycholinguist..

[41] Michael S.E., Ratner N.B., Newman R. (2012). Verb comprehension and use in children and adults with Down syndrome. J. Speech Lang. Hear. Res..

[42] Cleave P., Bird E.K.R., Czutrin R., Smith L. (2012). A longitudinal study of narrative development in children and adolescents with Down syndrome. Intellect. Dev. Disabil..

[43] Laws G., Hall A. (2014). Early hearing loss and language abilities in children with Down syndrome. Int. J. Lang. Commun. Disord..

[44] Van Bysterveldt A., Westerveld M., Guillon G., Foster-Cohen S. (2012). Personal narrative skills of school-aged children with Down syndrome. Int. J. Lang. Commun. Disord..

[45] Marini A., Martelli S., Gagliardi C., Fabbro F., Borgatti R. (2010). Narrative language in Williams syndrome and its neuropsychological correlates. J. Neurolinguist..

[46] Zampini L., Draghi L., Zanchi P. (2023). Developmental profiles in children and young adults with Alexander Disease. Dev. Neurorehabil..

[47] Pérez-García D., Flores R., Brun-Gasca C., Pérez-Jurado L. (2015). Lateral preference in Williams-Beuren syndrome is associated with cognition and language. Eur. Child Adolesc. Psych..

[48] Gonçalves O.F., Pinheiro A.P., Sampaio A., Sousa N., Férnandez M., Henriques M. (2011). Autobiographical narratives in Williams syndrome: Structural, process and content dimensions. J. Dev. Phys. Disabil..

[49] Montero I., León O. (2007). A guide for naming research studies in Psychology. Int. J. Clin. Health Psychol..

[50] Hayes A.F., Krippendorff K. (2007). Answering the call for a standard reliability measure for coding data. Commun. Methods Meas..

[51] Mayer M. (1974). Frog Goes To Dinner.

[52] Hessl D., Nguyen D.V., Green C., Chavez A., Tassone F., Hagerman R., Senturk D., Schneider A., Lightbody A., Reiss A. (2009). A solution to limitations of cognitive testing in children with intellectual disabilities: The case of fragile X syndrome. J. Neurodev. Disord..

[53] Salt Software. www.saltsoftware.com.

[54] Le K., Coelho C., Feinn R. (2023). Comprehension and Production in Traumatic Brain Injury. J. Speech Lang. Hear. Res..

[55] Meuris K., Maes B., Zink I. (2014). Evaluation of language and communication skills in adult key word signing users with intellectual disability: Advantages of a narrative task. Res. Dev. Disabil..

[56] Cao Y., Grace Kim Y. (2021). Is retell a valid measure of reading comprehension?. Educ. Res. Rev..

[57] Arfé B., Rossi C., Sicoli S. (2015). The Contribution of Verbal Working Memory to Deaf Children’s Oral and Written Production. The J. Deaf Stud. Deaf Educ..

[58] Carretti B., Motta E., Re A.M. (2016). Oral and Written Expression in Children with Reading Comprehension Difficulties. J. Lear. Disabil..

[59] Miles S., Chapman R.S. (2002). Narrative Content as Described by Individuals with Down Syndrome and Typically Developing Children. J. Speech Lang. Hear. Res..

