# Supplementary material for: How to Assess Oral Narrative Skills of Children and Adolescents with Intellectual Disabilities: A Systematic Review
Source: Behav Sci (Basel). 2024 Apr 10;14(4):308. doi: 10.3390/bs14040308 (PMC11047450; doi:10.3390/bs14040308)
Supplement: Supplementary file 1 [file behavsci-14-00308-s001.zip › Table S2.pdf]

Table S2. Main results of studies on narrative skills of individuals with ID.

**Study/***Tool*	**Level of analysis**	**Main results reported on narrative skills of individuals with ID**
Barton-Hulsey et al. (2017)<br>*FGTD (Generation)*	Macrostructure (Includes ISL)	On macrostructure, children with ID show stronger introduction, describing conflict/resolution and character development but show a weaker performance using mental state words, clear references and cohesive language. Macrostructure correlates with microstructural measures.
	Microstructure	Microstructural measures correlate with macrostructure and with reading comprehension skills.
Brown et al. (2018)<br>*Personal event (Generation)*	Macrostructure (Includes ISL)	Children with lower mental age, in particular those with ID (interaction), include fewer narrative coherence in personal reports than children with higher mental age. The narrative coherence correlates with testimonial accuracy measures.
Channell et al. (2015)<br>*FGTD/FOHO (Generation)*	Macrostructure (Includes ISL)	Children and adolescents with DS produce fewer episodic elements than mental age matched TD children only if MLU is not controlled. Children with DS and FXS show similar macrostructural performance.
	Microstructure	Both groups (DS and FXS) show similar MLU, adverb use and conjunction use. Participants with DS use less verbs than children with FXS and TD children.
Channell et al. (2020)<br>*FGTD/FOHO (Generation)*	Microstructure	In children with DS, MLU correlates with mental state language (density and diversity).
	ISL	Mental states language correlate with emotion knowledge and expressive vocabulary
Cleave et al. (2012)<br>*Bus Story Test (Retelling)*<br>*FOHO (Generation)*	Macrostructure (Includes ISL)	After 1 year without intervention, Children and adolescents with DS improve their information score and number of characters for the Bus story test, but not on number of events nor mental state words used. Children and adolescents with DS improve their story structure for the *Frog on His Own* on Number of characters, but not in Number of events or mental state words used
	Microstructure	Children and adolescents with DS did not show growth in syntactic complexity or narrative length over a 1-year period in the Bus story test. MA and comprehension skills predict narrative performance.
Diez-Itza et al. (2018)<br>*Cartoon scene (Retelling)*	Macrostructure	After an explicit intervention, students with WS improved their retelling of the story at macrostructural level in productivity but not in complexity (sequential order measures). Number of utterances predicted inversely the gains in macrostructure. Nonverbal IQ predicted macrostructural performance and gains in events, characters recalled and order of events.
	Microstructure	After an explicit intervention, students with WS improved their retelling of the story at microstructural level. Number of utterances predicted inversely the gains in microstructure productivity. Nonverbal IQ predicted microstructural performance in MLU and discourse markers.
Estigarribia et al. (2011)<br>*Bus story test (Retelling)*	Macrostructure (Includes ISL)	Narrative macrostructure in a retelling task is predicted by short-term memory and nonverbal mental age in children with ID (FXS, FXS-ASD and DS). Three groups with ID showed similar macrostructural performance. FXS-ASD group performed lower than FXS group.

Finestack et al. (2012) *FGTD (Generation)*	Macrostructure (Includes ISL)	Adolescents with FXS and DS showed a similar macrostructural performance assessed with NSS. Both groups showed a poorer macrostructural performance than TD MA-matched peers (Except for Introduction dimension in the case of DS).
	Microstructure	Adolescents with intellectual disabilities (ID) perform at a lower level than typically developing (TD) individuals on microstructural measures such as MLU and C-units. MLU mediates the observed differences on macrostructure between the ID and TD groups.
Gonçalves et al. (2011) *Open ended question (Generation)*	Macrostructure (Includes ISL)	Adolescents and young adults with WS produce personal narratives that are less coherent and complex than TD CA-matched peers. Both groups show similar performance in content diversity of narratives.
Hessling & Brimo (2019) *FGTD (Retelling)*	Macrostructure (Includes ISL)	Children with DS use episodic components in their narratives but do not use mental state words. Macrostructure (assessed with NSS) predict word-level reading and reading comprehension.
	Microstructure	Children with DS shows a restricted syntactic and semantic diversity and predominately use prepositional phrases and more verb than nouns. Microstructure (MLU, number of different words, and Narrative Assessment Protocol) predict word-level reading and correlate with reading comprehension.
Hettiarachchi (2016) *Peter and the cat (Retelling); Saman and the baby elephant (Generation)*	Macrostructure	After an intervention (Colorful semantics), children with ID showed better macrostructural performance (Content provided)
	Microstructure	After an intervention, children with ID showed a higher MLU and more complex sentences. The gain in both measures was higher in older children.
Hogan-Brown et al. (2013) *Bed full of cats (Generation)*	Macrostructure	Different etiologies showed similar macrostructural performance. MA show a main affect for macrostructure.
	Microstructure	An interaction between etiologies/diagnoses (FXS, FXS-ASD, ASD, DS) and nonverbal mental age predicted complex syntax in narrative microstructure. Not clear group differences in other microstructural measures were reported (such as MLU). Children with DS and ASD showed similar patterns. Nonverbal IQ mediate the relationship between genetic profile in FXS and language characteristics.
Jones (2013) *Thunder cake (Generation)*	Microstructure	Children WS use cohesive markers in narratives similarly to MA-matched children. Compared with CA-matched peers, children with WS show less referential cohesion. Children with WS produce similar grammatical error rates than TD children (CA or MA matched).
Laws & Hall (2014) *FWAY (Generation)*	Microstructure	MLUw correlates with CA and MA. MLUw correlates with Expressive Language (Assessed with the Reynell Development Language Scales) but this correlation is not significant after controlling by age (CA and MA) or Speech accuracy.
Marini et al. (2010) *Picnic and Cookie Theft and Flowerpot and Quarrel (Generation)*	Macrostructure	Macrostructural descriptions of stories by children with WS are less effective than those produced by TD children matched by mental age. A significant story effect was found for children with WS (Less informative in the two-cartoon-stories than in single-picture stories).
	Microstructure	Children with WS showed similar microstructural performance (phonological, lexical and syntactic skills) to MA-matched TD children.

Mastrogiuseppe & Lee (2017)<br>*Cartoon scene (Retelling)*	Microstructure	During narration, individuals with WS produce similar amount of clauses compared to MA-matched peers, but significant lower compared to CA-matched peers. Individuals with WS use fewer spatial words compared to the CA-matched group. As for gestures, individuals with WS produce more representative gestures during narration than MA-matched or CA-matched peers. Gestures produced by individuals with WS have a compensatory role during narration, (particularly in representing spatial content).
Micheal et al. (2012)<br>*Own elaboration story (Generation)*	Microstructure	Individuals with DS produced fewer words and shorter utterances in terms of MLU compared to the TD group (matched by receptive vocabulary age). DS group omitted verbs or other necessary elements (i.e., subject) in narrations more often than TD individuals. They perform significantly worse than TD group on the sentence memory task, while performing similarly to the TD group on all other measures of memory skills.
Neal et al. (2022)<br>*FGTD (Generation)*	Macrostructure	Female adolescents with FXS produce more complex narratives than males. Both female and males with FXS score best on Introduction and Mental states use, while poorer in other dimensions. Macrostructural performance in adolescents with FXS are predicted by language skills (vocabulary) and are correlated with literacy skills (written language).
	Microstructure	Males with FXS performed poorer than females with FXS on MLUm. Vocabulary skills predicted microstructural performance during narration.
Pérez-García et al. (2015)<br>*FWAY (Generation)*	Macrostructure (Includes ISL)	Individuals with WS with mixed-handedness showed worse performance in macrostructural performance than individuals with well-defined laterality (right or left).
Van Bysterveldt & Guillon (2014)<br>*Photographs (Generation)*	Macrostructure	Macrostructural score of personal narration correlate with reading measures (coefficients were not reported).
	Microstructure	Children with DS produced a very low MLUm during personal narration. Older children with DS used a greater number of different words in their personal narrative compared to younger ones. Microstructural measures were correlated with reading measures.
Zampini et al. (2023)<br>*NCT (Generation)*	Macrostructure (Includes ISL)	Children and youth with Alexander disease showed low performance in narrative macrostructure.
	Microstructure	Children and youth with Alexander disease showed low performance on narrative microstructure, but lower on macrostructural ones.
Zanchi et al. (2021)<br>*NCT (Generation)*	Macrostructure (includes ISL)	Children with DS showed a similar macrostructural performance compared to TD children (MA-matched or MLU-matched).
	Microstructure	Children with DS produce shorter and less complex sentences during narration than MA-matched children. No difference at the microstructural level, except for the verbal productivity, was found between children with DS and TD children MLU-matched.

ISL= Internal State Language; ID= Intellectual disability; DS= Down syndrome; FXS= Fragile X syndrome; WS= Williams syndrome; ASD= Autism spectrum disorder; TD= Typically developing; CA= Chronological age; MA= Mental age; IQ= Intelligence Quotient. MLU: mean length of utterance in words; MLUm: mean length of utterance in morphemes.
